# B cell-helping functions of gut microbial metabolites

**DOI:** 10.15698/mic2016.10.536

**Published:** 2016-09-23

**Authors:** Chang H. Kim

**Affiliations:** 1 Laboratory of Immunology and Hematopoiesis, Department of Comparative Pathobiology, Purdue University, West Lafayette, IN 47907, U.S.A.; 2 Weldon School of Biomedical Engineering, Purdue University, West Lafayette, IN 47907, U.S.A.; 3 Purdue Institute for Inflammation, Immunology and Infectious Diseases; Purdue University, West Lafayette, IN 47907, U.S.A.; 4 Purdue Center for Cancer Research; Purdue University, West Lafayette, IN 47907, U.S.A.

**Keywords:** commensal bacteria, dietary fiber, short-chain fatty acids, acetate, propionate, butyrate, B cells, antibodies, immunity

## Abstract

Commensal microflora profoundly affects the host immune system. It has long been observed that commensal bacteria enhance antibody production in the host by producing antigens for B cell receptors (BCR) and ligands for Toll-like receptors (TLR). We recently reported that the microbial metabolites short-chain fatty acids (SCFAs) regulate the metabolism and gene expression in B cells to promote antibody production (Kim *et al*. Gut Microbial Metabolites Fuel Host Antibody Responses. *Cell Host & Microbe*. 2016; 20(2):202-14). The B-cell helping function of SCFAs and its implication in the host immune system are discussed in this article.

Commensal bacteria comprehensively contribute to the health and diseases of the host. They contribute to host nutrition, energy harvest, metabolism, immune homeostasis, neuronal functions, and gut barrier function. Because bacterial groups are heterogeneous in their impact on the host, altered microbial composition, due to excessive expansion or reduction of particular bacterial groups (i.e. dysbiosis), is linked to pathophysiological conditions such as obesity, metabolic diseases, and inflammation. Importantly, commensal bacteria prepare the host immune system to respond to infections efficiently. This is important not only to fight infection but also to prevent chronic inflammatory responses caused by inadequate barrier functions or immunity. For example, gut microbiota stimulates B cells to increase antibody levels in the gut and blood circulation. Commensal bacteria also induce effector T cells such as Th17 cells and Th1 cells in the gut to increase intestinal T cell immunity. In addition, commensal bacteria promote immune tolerance by inducing suppressive immune cells such as regulatory T cells and tolerogenic myeloid cells.

Gut commensal bacteria ferment digestion-resistant carbohydrates in the colon. Soluble dietary fiber (DF) and digestion-resistant starches are such carbohydrates that reach the colon and are used by commensal bacteria to produce short-chain fatty acids (SCFAs). The Institute of Medicine recommends that adult men and women consume 25-38 g of DF each day. SCFAs are the major microbial metabolites in the colonic lumen. Early studies with ruminants established that SCFAs, particularly butyrate, are the major nutrients for colonic epithelial cells (also called colonocytes). Colonocytes and other cell types express transporters such as SLC16a1 and SLC5a8 to take up SCFAs into or export them out of cells for transportation. SCFAs function not only as nutrients but also activate cell-surface receptors such as GPR41, GPR43 and GPR109A, which are G-protein-coupled seven-transmembrane spanning receptors (GPCRs). GPCR activation by SCFAs on colonic epithelial cells is important to prepare gut epithelial cells to mount immune responses during infection. Moreover, neutrophils, enteroendocrine endothelial cells, and neuronal cells differentially express the GPCR receptors for SCFAs, which mediate, in part, immunological, neuroendocrinal or vascular regulatory functions of SCFAs. However, T and B cells do not express these receptors at significant levels.

Early research with rats revealed that DF intake increases IgA levels in the gut lumen. However, insoluble and non-fermentable DF such as cellulose does not have significant effects on IgA production, emphasizing the importance of soluble fermentable DF that produces SCFAs. Along with luminal IgA, serum IgA and IgG are increased by DF intake. The mechanism has been unclear until recently. Our group found that DF intake increases mucosal IgA and systemic IgG levels in mice and that this effect requires the role of gut microbiota. Commensal bacteria suppression with a minimally effective dose of antibiotics effectively decreased SCFA production and abolished the DF effect on antibody production. Moreover, all major SCFAs, such as acetate, propionate and butyrate, have B cell-helping activities, enhancing the production of IgA and IgG levels. Thus, this discovery establishes SCFAs as yet another group of B cell-helping microbial products (Figure 1).

**Figure 1:**
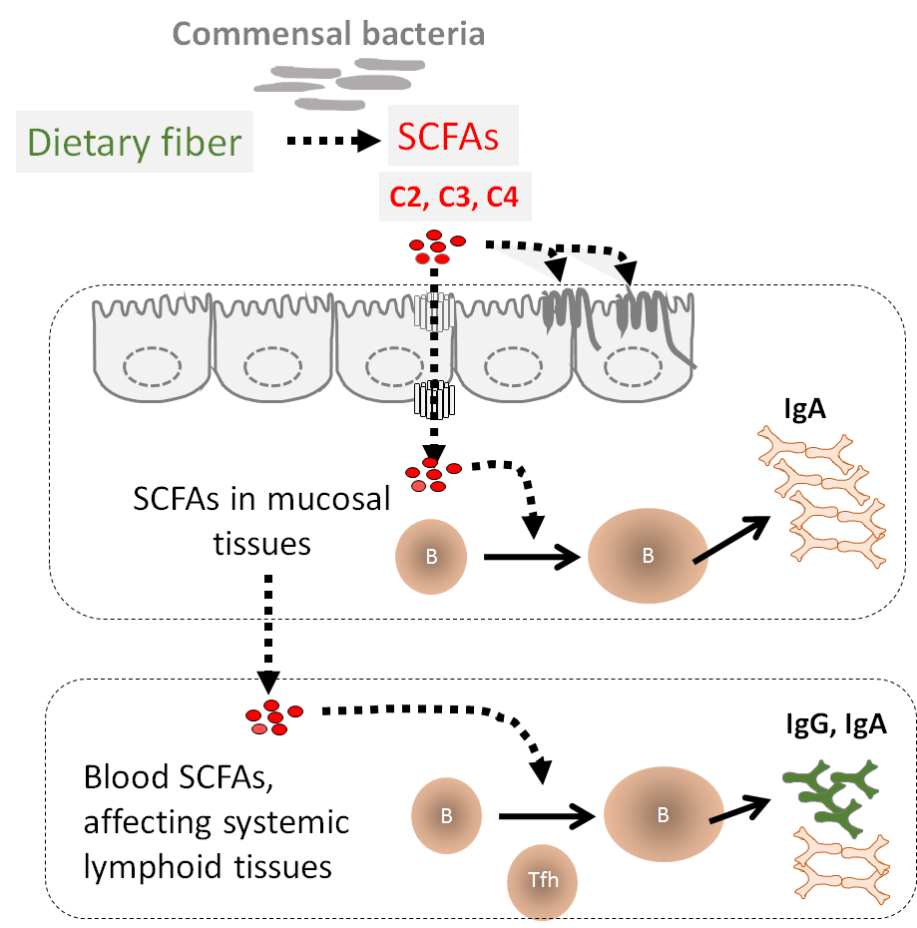
FIGURE 1: DF boosts antibody production through SCFAs. Soluble DF and digestion-resistant starches that reach the colon are fermented by commensal bacteria. This process generates SCFAs, which are absorbed by colonocytes. Some of the absorbed SCFAs are used by colonocytes but the remaining SCFAs are transported into gut tissues and reach the blood circulation. SCFAs in gut and associated-lymphoid tissues enhance B cell activation and plasma B cell differentiation, which are initiated by BCR-activating antigens, TLR ligands and/or co-stimulatory receptor ligands. This can lead to enhanced IgA production. The SCFAs that reach the blood circulation also affect B cell responses in systemic tissues. SCFAs boost the activation of B cells and production of IgG and IgA. SCFAs increase the synthesis of Acetyl-CoA, ATP and fatty acid in B cells to provide energy and building blocks required for plasma B cell differentiation. SCFAs also affect gene expression through their HDAC inhibitor activity to efficiently express genes for key regulators of B cell differentiation. The function of DF and SCFAs in helping B cells contributes to overall host immunity during the steady state and infection by pathogens.

How, in the world, do SCFAs enhance antibody production? We found an answer for this question in their effects on cellular metabolism. We observed that SCFAs increase Acetyl-CoA and mitochondrial oxidative phosphorylation for adenosine triphosphate (ATP) generation. SCFAs also increase glycolysis, which is regulated by the mechanistic target of rapamycin (mTOR) pathway. SCFAs increase the mTOR pathway in part by decreasing the cellular level of adenosine monophosphate (AMP) that stimulates AMP-activated kinase (AMPK). AMPK is a negative regulator of mTOR and, therefore, decreased AMPK activity by SCAFs leads to increased mTOR activity. Another process facilitated by SCFAs in B cells is lipid synthesis. Inhibition of each of these cellular pathways abolished the positive effect of SCFAs on B cell activation and antibody production in our study. Thus, SCFAs have a powerful influence on B cell metabolism, activation of which is critical to meet the high demand of energy and metabolic building blocks required for plasma B cell differentiation.

HDACs and histone acetyl transferases (HATs) reciprocally regulate histone acetylation and gene expression. The three major SCFAs, acetate, propionate and butyrate, are HDAC inhibitors. HDAC inhibition alters gene expression through increased histone acetylation in B cells. HDAC inhibition by SCFAs enhanced gene expression in the direction to promote plasma B cell differentiation in our study. Some of the SCFA-induced genes are *Xbp1*, *Irf4* and *Aicda*, which are key players in plasma B cell differentiation. Other genes regulated by SCFAs are those involved in cellular metabolism and antibody production.

Rather than initiating B cell activation on their own, SCFAs boost B cell responses to well-known B cell activators that activate BCR, TLR, and/or CD40. This indicates that SCFAs work together with various other B-cell stimulating microbial and host factors. In this regard, SCFAs increase homeostatic antibody responses to commensal flora as evidenced by increased IgA coating of gut bacteria. Moreover, SCFAs increased antibody responses to pathogens during *Citrobacter rodentium* infection. In line with the observation, the mice fed with DF-deficient diet were more susceptible to *C. rodentium *infection than the mice fed DF-containing control diet. This indicates that DF and SCFAs enhance immunity to prevent and clear infection by pathogens.

A number of questions arise regarding the function of DF and SCFAs in enhancing antibody production. Certain commensal bacteria ferment DF and, therefore, these bacteria can be enriched in high DF conditions. It is an intriguing question if the DF-enriched commensal microflora has any effects on antibody production. Moreover, increased production of SCFAs decreases pH levels in the gut lumen and this can affect microflora. In fact, it has been reported that DF alters gut microflora composition, increasing certain bacterial operational taxonomic units including the *Bacteroidetes* phylum. Currently, it remains to be determined if these bacteria are responsible for the increased B cell function in DF- or SCFA-supplemented animals. In a manner similar to the SCFA effect, probiotics such as* Lactobacillus* and* Bifidobacteria* increase intestinal IgA and systemic IgA and IgG production. It is not clear to date if SCFAs affect IgA production through gut epithelial cells. SCFAs stimulate epithelial cells and change their gene expression. Moreover, SCFAs increase the production of certain B cell-activating effector cytokines by epithelial cells. The importance of this pathway needs to be interrogated with further investigations.

SCFAs also regulate T cells. It has been documented that SCFAs increase colonic regulatory T cells that produce IL-10. SCFAs also promote the differentiation of effector T cells that produce IFN-γ and/or IL-17. Interestingly, SCFAs increased the numbers of follicular T helpers (Tfh cells), which promote germinal center responses in secondary lymphoid tissues. Similar to the function of Tfh cells, DF increases germinal center responses during infection. This function in increasing Tfh cells and germinal center responses is a potentially important mechanism for the B cell-helping functions of DF and SCFAs.

The recent progress in research indicates that SCFAs, initially known for their roles in supporting the integrity and inhibiting HDACs in colonocytes, are direct regulators of the adaptive immune cells, such as B and T cells. Beyond their function in suppressing inflammatory diseases and maintaining gut barrier immunity, SCFAs are key regulators of adaptive immunity. Particularly, their role in boosting antibody production has many ramifications in preventing and fighting infection by pathogens.

